# Effect of bacterial growth rate on bacteriophage population growth rate

**DOI:** 10.1002/mbo3.558

**Published:** 2017-12-01

**Authors:** Dominik Nabergoj, Petra Modic, Aleš Podgornik

**Affiliations:** ^1^ Center of Excellence for Biosensors Instrumentation and Process Control ‐ COBIK Ajdovščina Slovenia; ^2^ Faculty of Chemistry and Chemical Technology University of Ljubljana Ljubljana Slovenia

**Keywords:** bacterial growth rate, bacteriophage population growth rate, chemostat, *E. coli* K‐12, phage growth parameters, phage T4

## Abstract

It is important to understand how physiological state of the host influence propagation of bacteriophages (phages), due to the potential higher phage production needs in the future. In our study, we tried to elucidate the effect of bacterial growth rate on adsorption constant (δ), latent period (L), burst size (b), and bacteriophage population growth rate (λ). As a model system, a well‐studied phage T4 and *Escherichia coli* K‐12 as a host was used. Bacteria were grown in a continuous culture operating at dilution rates in the range between 0.06 and 0.98 hr^−1^. It was found that the burst size increases linearly from 8 PFU·cell^−1^ to 89 PFU·cell^−1^ with increase in bacteria growth rate. On the other hand, adsorption constant and latent period were both decreasing from 2.6∙10^‐9^ ml·min^−1^ and 80 min to reach limiting values of 0.5 × 10^‐9^ ml·min^−1^ and 27 min at higher growth rates, respectively. Both trends were mathematically described with Michaelis–Menten based type of equation and reasons for such form are discussed. By applying selected equations, a mathematical equation for prediction of bacteriophage population growth rate as a function of dilution rate was derived, reaching values around 8 hr^−1^ at highest dilution rate. Interestingly, almost identical description can be obtained using much simpler Monod type equation and possible reasons for this finding are discussed.

## 1 INTRODUCTION

Phages represent the most numerous and remarkably diverse organisms on Earth (Ackermann & Prangishvili, 2012; Clokie, Millard, Letarov, & Heaphy, 2011). Total number of phages on our planet has been estimated to be in the range of 10^30^–10^32^ (Brüssow & Hendrix, 2002), and surprisingly high concentration of phages (2∙10^8^ PFU ml^−1^) were detected in the samples from unpolluted lake water (Bergh, Børsheim, Bratbak, & Heldal, 1989). As natural killers of bacteria, phages are nowadays regaining attention due to alarming widespread emergence of bacteria resistant to majority of antimicrobial agents (Spellberg et al., 2008). Antibiotic resistance represents one of the biggest threats to global health, food safety, and development nowadays. Moreover, a growing number of infections are becoming harder to cure as the antibiotics are losing their efficacy. Consequently, antibiotic resistance leads to higher medical costs, longer hospital stays, and increased mortality (Roca et al., 2015). In 2014, the WHO declared antimicrobial resistance as a global health security threat (Prestinaci, Pezzotti, & Pantosti, 2015). Due to an alarming antibiotic crisis, phages could represent an interesting alternative to antibiotics. With growing awareness of significant influence of phages on environment, it is very important to study phage‐host interactions under unfavorable conditions that are usually occurring in natural environments. The advantages of antibacterial effect of phages have already been recognized and bacteriophage therapy was used in various fields such as veterinary medicine (Atterbury, 2009), agriculture (Jones et al., 2012), food industry (García, Martínez, Obeso, & Rodríguez, 2008), and also in human medicine (Abedon, Kuhl, Blasdel, & Kutter, 2011). In addition to the bacteriophage therapy, phages can also be used in clinical diagnostics (Schofield, Sharp, & Westwater, 2012), applied as vehicles for vaccines delivery or as potential carriers of therapeutic genes (Haq, Chaudhry, Akhtar, Andleeb, & Qadri, 2012). Also phage display, as a technique for the study of protein‐protein, protein‐peptide and protein‐DNA interactions, is possible thanks to existence of phages (Bazan, Całkosiński, & Gamian, 2012). Due to versatility of applications and consequently potential higher phage production needs in the future, it is important to understand how physiological state of the host influence propagation of phages. Phages are nowadays still being propagated old‐fashioned way in shaking flasks or bioreactors as a batch process. Consequently, many studies with phages are being made using the host cells that are growing exponentially, although vast majority of phage‐host interactions in nature are not occurring among the phages and exponentially growing host cells. However, pharmaceutical manufacturing is nowadays changing the trend from a batch to continuous production (Jungbauer, 2013; Lee et al., 2015). Thus, we can speculate that phage production for bacteriophage therapy will follow the same path in the future and new knowledge in this field is required (Podgornik, Janež, Smrekar, & Peterka, 2014). Chemostat is a bioreactor to which fresh medium is continuously added, while spent medium containing microorganisms, unconsumed nutrients and metabolic end products is removed at the same rate in order to keep the working volume constant (Novick & Szilard, 1950). One of its main advantages is that enables to tune microorganism specific growth rate and by this its physiology. Simply by changing the rate at which fresh medium is added, the specific growth rate of the microorganism adjusts spontaneously to equalize dilution rate. By this, different physiological state of the bacteria can be reproducibly achieved and since it remains constant over time, detailed analysis of various parameters can be performed (Ziv, Brandt, & Gresham, 2013). Because of that, chemostat experiments can provide an important insight into effect of bacterial physiological state on the phage propagation process and an interesting information also for continuous production. It has been already described that concentration of bacteria and bacterial physiological state have a significant influence on propagation of phages (Abedon, Herschler, & Stopar, 2001; Golec, Karczewska‐Golec, Łoś, & Węgrzyn, 2014; Hadas, Einav, Fishov, & Zaritsky, 1997; Middelboe, 2000; You, Suthers, & Yin, 2002). After irreversible adsorption of phages to the host receptors, phage DNA is transferred to bacterial cytoplasm where different developmental mechanisms such as lytic or lysogenic cycle, pseudolysogeny or carrier state can begin. In our case, obligately lytic phage T4 was chosen, since only lytic phages are recommended for bacteriophage therapy (Sulakvelidze, Alavidze, & Morris, 2001). Hadas and colleagues showed that propagation of phage T4 depends on growth conditions of its host, *E. coli* B/r (Hadas et al., 1997). In their experimental design, different media compositions were used to control the bacterial growth rate. On the other hand, Golec and colleagues used the same phage and bacterial strain as in our case (phage T4 and *E. coli* K‐12 MG1655, respectively), and different bacterial growth rates were achieved by varying the dilution rate in chemostat (Golec et al., 2014). It was revealed that latent period and burst size of phage T4 depend on bacterial growth rate. Also studies on different type of phages demonstrated that increase in bacterial growth rate shortens the eclipse and latent period, while burst size increases (Middelboe, 2000; You et al., 2002). In our study, we investigated how dilution rate, defining bacterial growth rate, affects adsorption constant (δ), latent period (L) and burst size (b) and consequently also bacteriophage population growth rate (λ). Later is defined as an increase in phages in medium over time and can be for a constant bacterial concentration (C) described by Equation (1) (Bull, 2006).

(1) $$\lambda \;=\;-m\;+\;\delta \cdot C(b\cdot e^{-L\cdot (d\;+\;\lambda )}-1)$$

Equation (1) also contains terms “m” and “d”, where “d” stands for intrinsic death rate of bacterial cells and “m” for death rate of free phage. We assumed that in our particular system term “m” can be neglected (being therefore equal to 0) due to long‐term stability of phage T4 at constant temperature (Bourdin et al., 2014). In chemostat cultures it is commonly also assumed that bacterial physiology adopts by specific growth rate to substrate limitation and cell death rate is found to be small, therefore commonly neglected, resulting in a well‐known equality that specific growth rate is equal to dilution rate. Therefore, we assumed that also term “d” is small enough to be neglected. Equation (1) then simplifies into Equation (2) (Podgornik et al., 2014).

(2) $$\lambda \;=\;\delta \cdot C(b\cdot e^{-L\cdot \lambda }-1)$$

Criteria of constant bacterial concentration are always met in chemostat where wide range of different bacterial growth rate is easily obtained (Ziv et al., 2013). In our case, *E. coli* K‐12 was grown in chemostat operating at dilution rates in the range between 0.06 to 0.98 hr^−1^. All the experiments for determination of adsorption constant, latent period and burst size were performed at each dilution rate once the steady state was achieved. Each phage growth parameter was mathematically described as a function of dilution rate and the results were used to estimate the bacteriophage population growth rate by Equation (2). Correlation between the bacteriophage population growth rate and the dilution rate was also obtained.

## 2 MATERIALS AND METHODS

### 2.1 Growth conditions of bacterial and phage strains

Phage T4 (DSM 4505) and *Escherichia coli* K‐12 MG1655 strain (DSM 18039) were used in all experiments. Bacterial cultures for phage titer determination and initial inoculation of bioreactor were prepared in laboratory flasks in low salt Lysogeny Broth (LB) (10.0 g tryptone, 5.0 g sodium chloride, 5.0 g yeast extract and distilled water to 1 L, pH 7) and incubated at 37°C overnight (Sambrook & Russell, 2001). LB medium was also used for growth of continuous culture of bacteria in chemostat. Bioreactor was a glass vial with 25 ml working volume. Stirring in bioreactor was achieved by a magnetic stirrer at 350 rpm. Compressed air was supplied with a flow rate of 2 L·min^−1^ through 0.22 μm filter to the glass bottle with a fresh medium. The fresh medium saturated with air was continuously supplied from 2 L glass bottles by silicone tubings to the bioreactor and the inlet and outlet flow rate was controlled by a single peristaltic pump (MiniPump, ShenChen). The whole experimental setup, including glass bottle with fresh medium, bioreactor, and silicone tubings, was autoclaved and assembled under sterile conditions. All the cultivations were performed in the incubator at 37°C. In order to achieve steady state, chemostat was operating for minimum of 16–96 hr (at least 8 generations) (Ziv et al., 2013), before the experiments for adsorption constant, latent period and burst size determination were performed. Bacterial concentration in chemostat was monitored on‐line through optical density (600 nm) by optical sensor to determine when steady state was achieved. Chemostat cultures reached steady state concentration of approximately 3 × 10^8^ CFU·ml^−1^ for all selected dilution rates. The dilution rates, being identical to the bacterial growth rates, were as follows: 0.06, 0.13, 0.26, 0.50, 0.60, 0.73, 0.82, and 0.98 hr^−1^.

### 2.2 Phage titer determination

The concentration of phages (PFU·ml^‐1^) was determined using standard double agar overlay plaque assay (Kropinski, Mazzocco, Waddell, Lingohr, & Johnson, 2009). In our case, double‐layer LB agar plastic Petri dishes with 90 mm diameter were used. Five milliliters of LB with 0.7% agar (w/v) was mixed with 100 μl of overnight bacterial culture and then poured on LB agar plate with 1.4% agar (w/v). Dilutions of phage samples were prepared in SM buffer (1 g gelatin, 5.8 g NaCl, 2 g MgSO_4_∙7H_2_O, 50 ml 1 mol/L Tris‐HCl (pH 7.5), distilled water to 1 L). Ten microliters of each dilution in triplicates were dropped on a bacterial lawn. LB agar plates were incubated at 37°C overnight and plaques were enumerated after approximately 16–18 hr of incubation.

### 2.3 Adsorption constant determination

Adsorption constant for each dilution rate was determined from three chemostat experiments according to standard protocol described elsewhere (Hyman & Abedon, 2009). Briefly, 1 ml of stabilized *E. coli* culture (*E. coli* culture collected from the chemostat outflow once the steady state had been achieved) was transferred to a 1.5 ml Eppendorf tube. Phage solution of the same temperature was added to the stabilized *E. coli* culture transferred from the chemostat to achieve multiplicity of infection (MOI) of 0.1, shortly mixed and incubated at 37°C without agitation. Samples of 50 μl were taken after 1, 2, 3, 4, 5, 6, 7, 8, 10, 15, and 20 min of incubation and transferred to new tubes containing 950 μl of SM buffer with 5 drops of chloroform. After gently mixing, the tube was incubated on ice for 10 min before the phage titer determination. Chloroform, ice and 20x dilution were used to kill the bacterial cells thus effectively removing also infected cells (Brown, 1956) and to slow down binding of phages and further adsorption (Kropinski, 2009), respectively. Initial concentration of phages (the starting concentration of phages at the time of infection) was determined in a sample prepared by the same volume of phages and medium but without bacteria. Initial concentration of bacteria (CFU·ml^−1^) was determined in triplicates using CFU assay. The adsorption constant was calculated from the slope of logarithm of free phages versus time and initial concentration of bacteria as described elsewhere (Hyman & Abedon, 2009).

### 2.4 Latent period and burst size determination

Latent period and burst size were determined by performing one‐step growth protocol in three chemostat experiments for each dilution rate (Golec et al., 2014; Hyman & Abedon, 2009). One milliliter of stabilized *E. coli* culture was transferred into a 1.5 ml Eppendorf tube. Phage solution of the same temperature was added to the stabilized *E. coli* culture transferred from the chemostat to obtain MOI of 0.1, the tube was then shortly mixed and incubated at 37°C without agitation. After 5 min, unadsorbed phages were removed by centrifugation (8000 g for 1 min), infected cells were resuspended in 1 ml of fresh LB medium (37°C) and 125 μl of infected cells were immediately transferred back to the 25 ml chemostat (200x dilution). The remaining volume of infected cells in the Eppendorf tube (unadsorbed phages were removed by centrifugation) was used to determine the number of infected cells. The same 200x dilution of infected cells as in the chemostat was prepared in fresh LB medium (37°C) and samples for phage titer determination were collected. Each sample (100 μl) was divided into two parts. The first 50 μl of the sample was added to the tube containing 950 μl of SM buffer with 5 drops of chloroform to kill bacteria, and by this prevent phage multiplication. After gently mixing, the tube was incubated on ice for 10 min before starting the phage titer determination procedure. The second 50 μl of the sample was added to the tube containing only 950 μl of SM buffer and immediately after short mixing, the phage titer determination was performed. Initial concentration of phages and bacteria was determined as described in adsorption constant determination section. Concentration of remaining unadsorbed phages after centrifugation was estimated from samples with chloroform. The number of infected cells (I_0_) was determined by subtracting the concentration of unadsorbed phages from the phage titer of samples without chloroform. In parallel, the samples for phage titer determination (50 μl) were collected from the chemostat outflow every 5–10 min from the start of infection (time when phage and bacteria were mixed together), depending on the dilution rate, for maximum 180 min. Samples were collected and treated as described above for samples without chloroform. Latent period was determined from the samples without chloroform, collected from the chemostat outflow, as the time from infection to the initial rise of phage titer. Burst size is defined as the number of phages released from each infected cell. For determination of burst size one should know the amount of formed phages during rise period and the amount of infected cells as well. Number of formed phages was calculated as a difference in phage titer at the beginning and end of rise period. Amount of infected cells present in bioreactor at the beginning of rise period is different from initial infected cell number (I_0_), since the infected cells are continuously washed out of the bioreactor what has to be taken into account. Assuming that infected cells do not multiply or lyse from infection till rise period, chemostat mass balance of infected cells can be written as:

(3) $$\frac{\mathrm{dI}}{\mathrm{dt}}=-D\cdot I$$

or in its integrated form:

(4) $$I=I_{0}\cdot e^{-D\cdot t}$$

Burst size was calculated by dividing number of phages formed during rise period with the estimated number of infected cells (I) present in the bioreactor at the latent period time as described elsewhere (Hadas et al., 1997). Due to short time of rise period, the decrease in released phages due to washing was neglected. Detailed information and example of calculations for latent period and burst size determination in the chemostat is provided in the Figure S1, Table S1. Based on Equation (4) it can be calculated that decrease in infected cells is from 7 to 29% (Table S2) for implemented experimental conditions and should not be neglected.

### 2.5 RNA/protein ratio determination

To determine the RNA/protein ratio, a general protocol using the TRIzol^TM^ reagent (Invitrogen) was performed according to the instructions (Rio, Ares, Hannon, & Nilsen, 2010) to purify RNA, DNA, and protein from the same sample (Chomczynski & Sacchi, 1987). Briefly, the sample of stabilized *E. coli* cells was collected from the chemostat outflow and diluted in HyClone^TM^ water to 0.25 ml containing 1∙10^7^ CFU·ml^−1^ cells. Diluted cells (0.25 ml) were transferred to a 2 ml Eppendorf tube and supernatant was removed by centrifugation (10,000*g* for 10 min); 0.75 ml of TRIzol^TM^ reagent was added to the pellet and homogenized by pipetting. The sample was incubated at room temperature for 5 min and then 0.15 ml of chloroform was added. The sample was further incubated at room temperature for additional 3 min and separated afterward by centrifugation (12,000*g* for 15 min at 4°C) into lower red phenol‐chloroform phase, interphase, and a colorless upper aqueous phase. The aqueous phase containing RNA was transferred to a new tube by angling the tube at 45° and pipetting. A quantity of 0.375 ml of isopropanol was added to the aqueous phase and the sample was incubated for 10 min. The supernatant was removed by centrifugation (12,000*g* for 10 min at 4°C) and pellet was resuspended in 0.75 ml of 75% ethanol. After short mixing, the supernatant was again removed by centrifugation (7,500 g for 5 min at 4°C) and the RNA pellet was air‐dried for 5 min. The RNA pellet was resuspended in 50 μl of RNase‐free water and the sample was incubated in water bath at 55°C for 12 min. RNA samples were quantified by absorbance (260 nm) using the NanoDrop^TM^ spectrophotometer according to the instrument instructions. On the other hand, proteins were isolated from the lower red phenol‐chloroform phase; 0.225 ml of 100% ethanol was added to the phenol‐chloroform phase and mixed by inverting the Eppendorf tube several times. The sample was incubated at room temperature for 3 min and then the DNA was pelleted by centrifugation (2,000*g* for 5 min at 4°C). The phenol‐ethanol supernantant was transferred to a new Eppendorf tube. 1.125 ml of isopropanol was added and sample was incubated at room temperature for 10 min. The supernatant was removed by centrifugation (12,000*g* for 10 min at 4°C) and pellet was resuspended in 1.5 ml of 0.3 mol/L guanidine hydrochloride in 95% ethanol. The sample was incubated at room temperature for 20 min. The supernatant was removed by centrifugation (7,500*g* for 5 min at 4°C) and pellet was again resuspended in 1.5 ml of 0.3 mol/L guanidine hydrochloride in 95% ethanol. Washing step was repeated twice and then pellet was resuspended in 2 ml of 100% ethanol and mixed by vortexing. The sample was incubated at room temperature for 20 min and then the supernantant was removed by centrifugation (7,500*g* for 5 min at 4°C). The pellet was air‐dried for 10 min and then resuspended in 200 μl of 1% SDS in 20 mmol/L Tris buffer, pH = 7.5). The sample was incubated in water bath at 55°C for 10 min. Insoluble material was removed by centrifugation (10,000*g* for 10 min at 4°C) and the supernatant was transferred to a new tube. Protein samples were quantified by absorbance (280 nm) using the NanoDrop^TM^ spectrophotometer according to the instrument instructions. RNA/protein ratio was calculated for each dilution rate and the correlation between the RNA/protein ratio and dilution rate was obtained.

## 3 RESULTS

Adsorption constant (δ), latent period (L), and burst size (b) represent phage growth parameters which altogether define bacteriophage population growth rate (λ). Various researchers showed that a growth rate of bacteria influence phage growth parameters, especially changes in latent period and burst size (Abedon et al., 2001; Golec et al., 2014; Hadas et al., 1997; Middelboe, 2000; You et al., 2002). In this study, we first analyzed the effect of dilution rate in the range between 0.06 and 0.98 hr^−1^ of *E. coli* K‐12 chemostat cultures on phage growth parameters of phage T4. Table 1 contains results of phage growth parameters for each dilution rate studied. Latent period decreased when the dilution rate increased and reached a minimum of 27 min. In contrast, burst size increased with increasing dilution rate. Interestingly, adsorption constant remained almost constant (0.5 × 10^−9^ ml·min^−1^) between the dilution rates of 0.60–0.98 hr^−1^, nevertheless it started to increase up to 2.6 × 10^−9^ ml·min^−1^ when the dilution rate decreased down to the lowest dilution rate studied. Experimental data for adsorption constant, latent period and burst size as functions of dilution rate were fitted by Equation (5), 6, and 7, respectively.

**Table 1:** Results of phage growth parameters for each dilution rate studied^a^

Dilution rate (hr^−1^)	Adsorption constant (10^−9^ ml min^−1^)	Latent period (min)	Burst size (PFU per 1 cell)
0.06	2.6 ± 0.24	80 ± 4	8 ± 2
0.13	2.0 ± 0.12	60 ± 4	13 ± 3
0.26	1.1 ± 0.19	41 ± 1	20 ± 5
0.50	0.81 ± 0.04	36 ± 4	33 ± 6
0.60	0.53 ± 0.04	31 ± 3	59 ± 3
0.73	0.42 ± 0.07	29 ± 3	66 ± 7
0.82	0.50 ± 0.05	27 ± 1	75 ± 4
0.98	0.52 ± 0.04	27 ± 1	89 ± 4

a Three chemostat experiments for each dilution rate were performed to determine phage growth parameters. Results are shown as average values ± SD.

(5) $$\delta =\delta_{\max}-\delta^{\prime }\cdot \frac{D}{K_{\mathrm{ads}}+D}$$

(6) $$L=\frac{K_{\mathrm{lat}}+D}{\frac{1}{L_{\min}}\cdot D}$$

(7) b=k·D

When equations (5), (6) and (7) are inserted into Equation (2), Equation (8) is obtained:

(8) $$\lambda =\left[\delta_{\max}-\delta^{\prime }\cdot \frac{D}{K_{\mathrm{ads}}+D}\right]\cdot C\left(\left[k\cdot D\right]\cdot e^{-\left[\frac{K_{\mathrm{lat}}+D}{\frac{1}{L_{\min}}\cdot D}\right]\cdot \lambda }-1\right)$$

**Figure 1:**
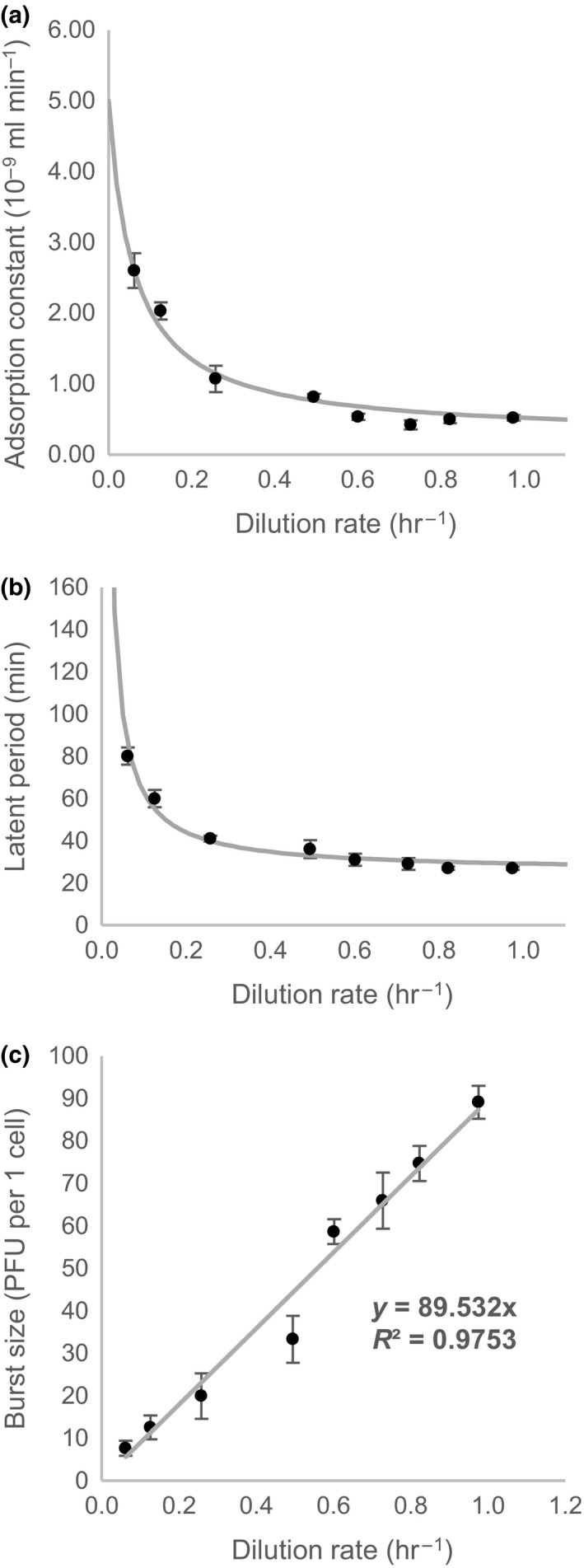
Phage growth parameters as a function of dilution rate. (a) Adsorption constant, (b) latent period and (c) burst size, respectively. Black dots with error bars represent experimental data, while solid gray lines represent best fit of equations (5), 6, and 7, respectively

**Figure 2:**
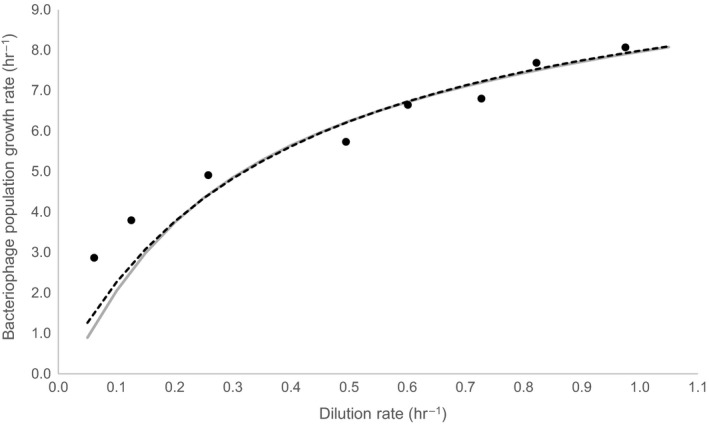
Bacteriophage population growth rate as a function of dilution rate. Black dots represent results of bacteriophage population growth rate calculated for each dilution rate by inserting experimentally determined phage growth parameters in Equation (2). Solid gray line represents values of bacteriophage population growth rate obtained by plotting Equation (1) using parameters from Table 2, while black dotted line represents values of bacteriophage population growth rate calculated by the best fit of Equation (8)

**Figure 3:**
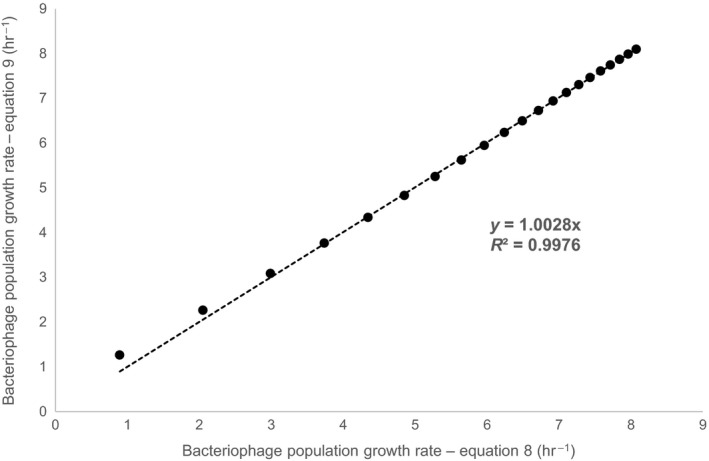
Comparison of bacteriophage population growth rate determined by Equation (1) and 9. Black dots represent the values of bacteriophage population growth rate determined by both equations, while black dotted line represents the linear correlation (*R* ^2 ^= .9976)

Equation (5) reflects certain analogy to equation of Langmuir adsorption isotherm, Equation 6 represents a reciprocal Michaelis–Menten equation and Equation (7) is a simple linear equation. Reasons for such selection of equation type are explained in Discussion section. Figure 1 shows experimental data for each phage growth parameter as a function of dilution rate and fitted with appropriate mathematical equation. Fitted equations coefficients are presented in Table 2. Bacteriophage population growth rate was calculated for each dilution rate by inserting experimentally determined burst size, latent period and adsorption constant in Equation (2). Furthermore, parameters from Table 2 were used to plot Equation (1). Figure 2 and Table S3 represents the results of bacteriophage population growth rate as a function of dilution rate. When the growth rate increased, bacteriophage population growth rate also increased. The Equation (8) (see below), which represent a form of Monod equation, was used to describe in a simple manner the change in bacteriophage population growth rate due to dilution rate and very similar trend as with Equation (1) was obtained (Figure 2) demonstrated also by almost perfect linear correlation (*R* ^2 ^= .9976) when compared (Figure 3).

**Table 2:** Fitting equation coefficients

Adsorption constant			Latent period			Burst size		
δ_max_	5.00 × 10^‐9^	ml·min^−1^	K_lat_	0.145	hr^−1^	k	89.532	PFU cell^−1^ hr
δ’	4.75 × 10^‐9^	ml·min^−1^	L_min_	25.5	min			
K_ads_	0.060	hr^−1^						

(9) $$\lambda =\frac{\lambda_{\max}\cdot D}{K_{\lambda }+D}$$

Parameters of Equation (8) (λ_max_ and K_λ_) were defined to be 11.1 hr^−1^ and 0.39 hr^−1^, respectively.

## 4 DISCUSSION

To investigate the effect of bacterial growth rate on bacteriophage interaction and bacteriophage population growth rate a well‐studied phage T4 and *E. coli* K‐12 as a host was used. *E. coli* K‐12 was grown in a continuous culture in a chemostat, where dilution rate determines the growth rate after the steady‐state is achieved. All our experiments were performed in LB medium, which is complex but not well‐defined medium that possess limitations due to low amount of carbohydrates and other utilizable carbon sources such as peptides and free amino acids important for *E. coli* growth (Sezonov, Joseleau‐Petit, & D'Ari, 2007; Wang & Koch, 1978) but also low amount of divalent cations (Ca^2+^ and Mg^2+^) (Wee & Wilkinson, 1988), especially important for binding of phage T4 to the host (Kutter et al., 1994). Unfortunately, we cannot say which of these components of LB medium represent a limiting factor for *E. coli* growth in our case. However, since almost perfect linear correlation (R^2^ = 0.9831) between RNA/protein ratio and dilution rate was observed (Figure S2 ) and RNA/protein ratio was previously demonstrated to be proportional to the specific growth rate (Scott, Gunderson, Mateescu, Zhang, & Hwa, 2010), there is an indication that although the not‐well defined LB medium was used, chemostat dilution rate directly affected bacterial physiological state. The experiments of phage growth parameters, namely adsorption constant, latent period and burst size, were performed for a wide range of dilution rates and obtained trends for each phage growth parameter were fitted with equations (5), (7). Form of selected equations used for fitting was chosen to reflect some relevant underlying biologic or physical mechanism. When the dilution rate increased, the latent period decreased and the burst size increased (Figure 1b & c), so both parameters changed in the similar fashion as it has been already described in the literature (Abedon et al., 2001; Golec et al., 2014; Hadas et al., 1997). It is interesting, that burst size linearly (*R* ^2 ^= .9753) increased with increasing dilution rate in the studied range (Figure 1c). This might be explained by special mode of response of phage T4. Recently, Bryan and colleagues described an interesting phenomena when *E. coli* cells in stationary phase were exposed to phage T4 infection (Bryan, El‐Shibiny, Hobbs, Porter, & Kutter, 2016). The authors explored the response of T4‐infected stationary phase cells to the addition of fresh nutrients certain time after the infection and new mode of response known as “hibernation” mode was discovered. “Hibernation” mode is a persistant but reversible dormant state in which the infected cells produce some phage enzymes, but halt phage development until appropriate nutrients become available before producing phage particles. This process might also explain observed burst size trend. We can consider cells in stationary phase and cells in growing at maximal growth rate as two extreme cases: in the first one “hibernation” mode is completely present (for all cells) while non‐existent during maximal growth rate. Chemostat force culture to “travel” between these two extremes (different specific growth rates defined by dilution rate). Assuming that during this “travel” ratio of cells entering “hibernation” mode proportionally increases. As cells where reversible “hibernation” mode is present do not produce phages under implemented conditions, it looks as that burst size decreases. Due to a proportionality, this would result in a linear relation between burst size and dilution rate, as our results do indicate. This hypothesis of course has to be verified with further work. On the other hand, our results might also be biased by “hibernation” mode due to experimental set‐up. During infection with phages, infected cells were after the centrifugation exposed to the fresh nutrients since they were resuspended in fresh LB medium for few seconds before being transferred back to the chemostat. This might burst phages from cells being in “hibernation”, what would minimize the differences in burst size of cells in different physiological state.

In contrast to linear increase in burst size with dilution rate, the latent period seems to converge toward limit value reaching a minimum of around 27 min for high dilution rates, an increase in threefold in comparison to value determined at the lowest dilution rate studied (Figure 1b). For mathematical description of this trend a reciprocal Michaelis‐Menton equation was used (Equation 6). There are two main reasons for this selection. First, the latent period should converge to the minimal latent period possible when the growth rate increases toward the maximum value which represents maximal specific growth rate. In our case, the minimal latent period of 25.5 min for wild‐type phage T4 was used in Equation 6, according to the literature data (Abedon et al., 2001). Second, when dilution rate approaches to 0 also substrate supply is cut. Because of that the growth rate is also approaching 0 hr^−1^, while latent period should approach to infinity as, in contrary, phages would be generated from nothing, violating therefore mass conservation law. This seems somehow to happen in recently discovered reversible “hibernation” mode of phage T4 (Bryan et al., 2016). Reciprocal Michaelis–Menten equation fulfills both assumptions and, in addition, limiting fitted value matches values reported in literature (Abedon et al., 2001) indicating that there might be some physiological origin for such trend. However, further work is needed to verify this hypothesis.

Finally, Equation (5) was selected to describe experimental data of adsorption constant as a function of dilution rate. The process of phage adsorption to the host cells is a physical process as it was already shown by Krueger, since phages can also adsorb to dead bacteria (Krueger, 1931). It was also shown that the rate of adsorption not only depends on host physiological state and cultural conditions but it is also influenced by a variety of non‐specific physical‐chemical factors such as temperature, pH, osmolarity, ionic strength, electrolyte requirements (divalent cations such as Mg^2+^ and Ca^2+^), adsorption cofactors (L‐tryptophan in the case of phage T4), and even mixing (e.g., motion within or of the adsorption medium) (Delbrück, 1940; Hyman & Abedon, 2009; Kutter et al., 1994; Marcó, Reinheimer, & Quiberoni, 2010; Mojica & Brussaard, 2014; Quiberoni, Guglielmotti, Binetti, & Reinheimer, 2004; Sillankorva, Oliveira, Vieira, Sutherland, & Azeredo, 2004). Since we only studied adsorption of phages and no further infection, where proton motive force is required for DNA translocation (Hu, Margolin, Molineux, & Liu, 2015), we can consider that adsorption constant mainly reflects physical adsorption, while physiology (different dilution rate in our case) is only important for cell wall properties, therefore characteristics of surface on which phages are adsorbed. In our case, when the dilution rate is approaching 0 hr^−1^, the extrapolated adsorption constant is approaching to the maximal value of 5∙10^‐9^ ml·min^−1^ (Figure 1a). The latter value is below the theoretical upper limit of adsorption constant (1∙10^−8^ ml·min^−1^), which was estimated from phage diffusivity and bacterial cell size and is approached when nearly all encounters of phages to the host result in adsorption (Schlesinger, 1932). Interestingly, adsorption constant increased by fivefold when dilution rate decreased from 0.6 to 0.06 hr^−1^, whereas it remained almost constant (0.5∙10^‐9^ ml min^−1^) between the dilution rates of 0.60–0.98 hr^−1^ (Figure 1a). These results are to some extent in contrast with the literature (Golec et al., 2014; Hadas et al., 1997). According to Hadas and colleagues, rates of T4 adsorption increased with increasing growth rate in richer media, but different growth rates of *E. coli* B/r were obtained by several modifications of the media composition (Hadas et al., 1997). In contrast to our experimental design, authors did not use chemostat to grow bacteria with well‐defined growth rate, but influenced the growth rate by modification of media composition. The same phage T4 as in our experiments, but different host strain (exponentially grown *E. coli* B/r) were used in their study. On the other hand, Golec and his colleagues performed similar experiments in chemostat as we did, using the same phage and the same bacteria strain (phage T4 and *E. coli* K‐12 MG1655, respectively), but different growth media (phosphate‐buffered minimal medium containing 10 g/L glucose) (Golec et al., 2014). It was reported that no significant differences in the efficiency of phage adsorptions on continuous cultures (growth rates from 0.3 to 0.033 hr^−1^) were found. Authors concluded that the bacterial growth rate has no significant impact on T4 adsorption in chemostat cultures. In our case, the differences in adsorption rates due to different dilution rates were clearly observed (Figure 1a). According to the results of adsorption constant in Figure 1a we believe that divalent cations Mg^2+^ and Ca^2+^ are not directly responsible for observed trend ‐ increase in adsorption constant when dilution rate was decreasing. Low dilution rate results in low medium concentrations of Mg^2+^ and Ca^2+^, being the lowest at the lowest dilution rate. As T4 requires Mg^2+^ and Ca^2+^ cations for the binding (Kutter et al., 1994), we would because of that expect the lowest adsorption constant at the lowest dilution rate, therefore trend just opposite that we experimentally observed. That is why we speculate that limitation of certain component in the medium cannot be directly responsible for observed trend but it can have an indirect impact. According to the literature, phage T4 adsorbs to the lipopolysaccharides (LPS) in the outer membrane in *E. coli* B/r, whereas T4 requires LPS and outer membrane porin protein C (OmpC) as well for proper phage receptor function in *E. coli* K‐12 (Henning & Jann, 1979; Rakhuba, Kolomiets, Dey, & Novik, 2010; Washizaki, Yonesaki, & Otsuka, 2016; Yu & Mizushima, 1982). The concentration of LPS was estimated at 10^6^ monomers of LPS per bacterial cell, the same value was determined for different strains (Smit, Kamio, & Nikaido, 1975; Washizaki et al., 2016). Moreover, it was shown that the number of LPS per cell remained constant although bacteria was exposed to starvation process (Walczak et al., 2012). On the other hand, the estimated concentration of OmpC is ten times lower than concentration of LPS, representing 10^5^ OmpC molecules per cell (Darcan, Ozkanca, & Flint, 2003; Lugtenberg & Van Alphen, 1983; Osborn & Wu, 1980). Because of that, one can assume that OmpC concentration actually determines phage adsorption. Liu and Ferenci studied the regulation of porin‐mediated outer membrane permeability by nutrient limitation in *E. coli* chemostat cultures (Liu & Ferenci, 1998). The authors demonstrated that the expression of OmpC is increased at low growth rates (from 0.3 to 0.1 hr^−1^) under glucose and ammonia limitation (threefold increase and fivefold increase, respectively). It was also shown that OmpC is expressed at higher level under anaerobiosis (Matsubara, Kitaoka, Takeda, & Mizuno, 2000; Nikaido, 2003). In our case, observed increased adsorption phenomenon of phage T4 on continuous culture of *E. coli* K‐12 at low dilution rates could be explained by the potential increase in OmpC receptors on the host under limitation conditions, which could provide additional binding sites for the unadsorbed phages. This manifests in faster adsorption rate due to higher number of successful collisions between phages and bacteria cells resulting in adsorption of phages. Rather constant value of OmpC at higher growth rates (Liu & Ferenci, 1998) should therefore preserve constant adsorption rate as observed experimentally in our case. At lower growth rate and consequently higher OmpC concentration on the cell surface also adsorption constant increases proportionally. There must be an upper limit, when the entire cell surface is available for binding, meaning that every collision results in adsorption, determined by Schlesinger (Schlesinger, 1932) to be equal to 1∙10^‐8^ ml·min^−1^. However, this value differs from bacteria and phage strain and commonly lower values are encountered (Denes, den Bakker, Tokman, Guldimann, & Wiedmann, 2015; Merabishvili et al., 2014; Moldovan, Chapman‐McQuiston, & Wu, 2007; Quiberoni et al., 2004), in our case it was predicted to be 5∙10^‐9 ^ml·min^−1^. Described mechanism also indicates a type of equation for description of adsorption constant. Starting from maximal value at lowest dilution rate, there should be a decrease in adsorption constant converging toward limiting value. As adsorption kinetics is related to adsorption, description with Langmuir adsorption isotherm equation seems to be reasonable (being however identical by the form to Michaelis‐Menten equation). Good fitting with experimental data was obtained (Figure 1a).

Based on above analysis one can also investigate effect of dilution rate on bacteriophage population growth rate. As one could anticipate, bacteriophage population growth rate increases with increasing dilution rate (Figure 2, Table S3) approaching its maximal value at maximal bacterial growth rate. Interestingly, bacteriophage population growth rate increased faster at low dilution rates while slower increase was observed at higher dilution rates. When dilution rate increased from 0.05 to 0.3 hr^−1^, 0.3 to 0.6 hr^−1^, and 0.6 to 0.9 hr^−1^, the bacteriophage population growth rate increased for 4 hr^−1^, 1.5 hr^−1^, and 1 hr^−1^, respectively. Based on equation (1) it is possible to predict bacteriophage population growth rate at defined dilution rate as shown in Figure 2. However, due to the structure of Equation (1) it is not possible to obtain its analytical solution, but it should be numerically solved for each dilution rate. Observed trend predicted by Equation (1) seems to resemble to Michaelis–Menten type of correlation and when implemented Equation (8), an excellent correlation between both data series was obtained (Figure 3). This rather interesting finding can also have a physiological rational. As phages are multiplied from bacteria, the latter can be considered as phage substrate and analogy with Monod description of microorganism specific growth rate as a function of substrate concentration can be drawn (Monod, 1949). Although this is a clear oversimplification it can nevertheless be used as a rational for a simple description of bacteriophage population growth rate at different growth rates of bacteria.

In this work, we tried to elucidate the effect of bacterial growth rate on bacteriophage population growth rate. The results clearly showed that bacterial growth rate has an important influence on all three phage growth parameters, namely adsorption constant, latent period and burst size, which together determine the bacteriophage population growth rate. In our case, bacteriophage population growth rate as a function of dilution rate was found to be accurately described by simple Monod equation. At this point, the question emerges if these observations are general or depend on the choice of phage‐host system. However, even if observed trends are system dependent, proposed approach enables design of phage production process and can therefore be generally implemented to estimate time needed for production of required amount of phages.

## CONFLICT OF INTEREST

None declared.
