# Supplementary material for: Effect of bacterial growth rate on bacteriophage population growth rate
Source: Microbiologyopen. 2017 Dec 1;7(2):e00558. doi: 10.1002/mbo3.558 (PMC5911998; doi:10.1002/mbo3.558)
Supplement: Supplementary file 1 [file MBO3-7-na-s001.docx]

**Supporting Information**

**Burst size and latent period determination in the chemostat**

Burst size and latent period determination in the chemostat are explained on the example of experimental data (dilution rate in the chemostat was 0.6 h^-1^). Latent period was determined from the samples without chloroform, collected from the chemostat outflow, as the time from infection to the initial rise of phage titer. In presented example, the latent period was estimated to be 33 minutes (Fig. S1). Burst size is defined as the number of phages released from each infected cell. For determination of burst size one should know the amount of formed phages during the rise period and the amount of infected cells as well. Number of formed phages was calculated as a difference in phage titer at the beginning and end of first rise period using samples without chloroform, collected from the chemostat outflow. In our example, the average phage titer from the beginning of rise period was 3.0∙10^3^ PFU mL^-1^, while average phage titer at end of rise period was 1.32∙10^5^ PFU mL^-1^ (Fig. S1). The difference in phage titers was calculated to be 1.29∙10^5^ PFU mL^-1^.


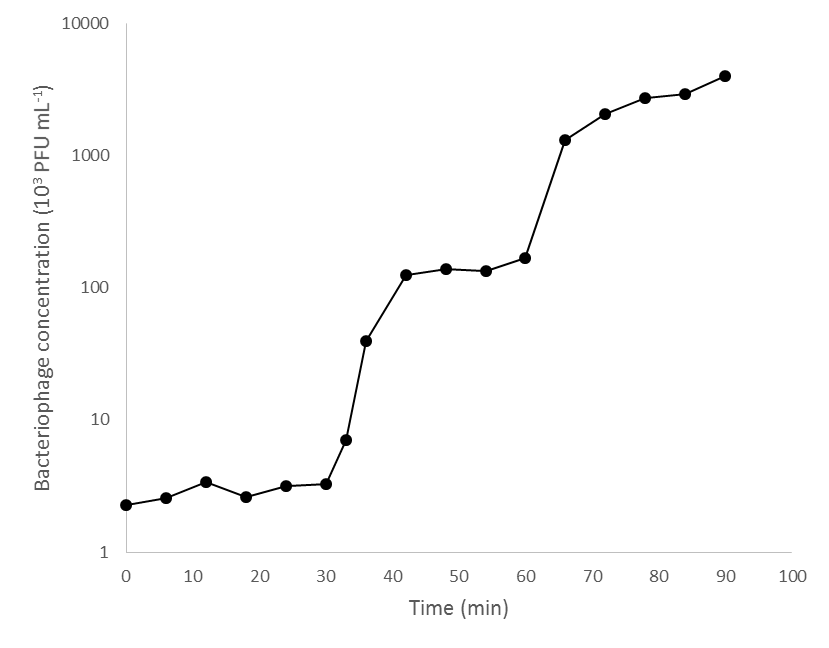


**Figure S1**. One-step growth experiment in the chemostat operating at dilution rate 0.6 h^-1^. Concentration of free phages (see below) was deducted from the concentration of phages determined in samples without chloroform, collected from the chemostat outflow. Black dots represent obtained results.

As described in the manuscript under section latent period and burst size determination, the remaining volume of infected cells in the Eppendorf tube (unadsorbed phages were removed by centrifugation) was used to determine the number of infected cells. Concentration of remaining unadsorbed phages after centrifugation was estimated from three samples with chloroform to be 1.3∙10^3^ PFU mL^-1^. The number of initial infected cells (I_0_) was determined by subtracting the concentration of unadsorbed phages from the phage titers of three samples without chloroform, and the value was estimated to be 3.0∙10^3^ PFU mL^-1^. Amount of infected cells (I) present in the bioreactor at the beginning of rise period was calculated using the equation 4 from the manuscript as it is shown in Table S1. Concentration of infected cells (I) was estimated to be 2.29∙10^3^ PFU mL^-1^ (value at the time of 27 min in Table S1), since the latent period was determined to be 33 mins and the infected cells were the first 6 minutes outside of the chemostat. In presented example, the concentration of infected cells in the chemostat decreased for 23.7 % at the time of latent period compared to the number of initial infected cells (Table S1). Burst size was calculated by dividing the number of phages formed during rise period (1.29∙10^5^ PFU mL^-1^) with the estimated number of infected cells (2.29∙10^3^ PFU mL^-1^) present in the bioreactor at the latent period time. The burst size for dilution rate 0.6 h^-1^ was determined to be 56.4 PFU cell^-1^. All the presented results of burst size and latent period in the manuscript were obtained using the described protocol.

| Table S1. Decrease of infected cells in the chemostat (dilution rate 0.6 h^-1^) with time | | | |
| --- | --- | --- | --- |
| Time (min) | Concentration of infected cells  (10^3^ PFU mL^-1^) | | Decrease in infected cells (%) |
| 0 | 3.00 | 0.0 | |
| 3 | 2.91 | 3.0 | |
| 6 | 2.82 | 5.8 | |
| 9 | 2.74 | 8.6 | |
| 12 | 2.66 | 11.3 | |
| 15 | 2.58 | 13.9 | |
| 18 | 2.50 | 16.5 | |
| 21 | 2.43 | 18.9 | |
| 24 | 2.36 | 21.3 | |
| 27 | 2.29 | 23.7 | |
| 30 | 2.22 | 25.9 | |
| 33 | 2.15 | 28.1 | |
| 36 | 2.09 | 30.2 | |
| 39 | 2.03 | 32.3 | |
| 42 | 1.97 | 34.3 | |

Table S2 includes the calculation of decrease of infected cells in the chemostat with time for studied range of dilution rates (the initial number of infected cells (I_0_) was set to be 3∙10^3^ PFU mL^-1^ in all cases). The results show that decrease in infected cells is from 7 to 29% for dilution rates from 0.06 to 0.98 h^-1^, respectively.

| Table S2. Decrease of infected cells in the chemostat with time for studied range of dilution rates^a^ | | | | | | | | | |
| --- | --- | --- | --- | --- | --- | --- | --- | --- | --- |
|  | Decrease in infected cells (%) | | | | | | | | |
| Time (min) \ Dilution rate (h^-1^) | 0.06 | 0.13 | 0.26 | 0.50 | 0.60 | 0.73 | 0.82 | 0.98 |  |
| 0 | 0.0 | 0.0 | 0.0 | 0.0 | 0.0 | 0.0 | 0.0 | 0.0 |  |
| 3 | 0.3 | 0.6 | 1.3 | 2.5 | 3.0 | 3.6 | 4.0 | 4.8 |  |
| 6 | 0.6 | 1.3 | 2.6 | 4.9 | 5.8 | 7.0 | 7.9 | 9.3 |  |
| 9 | 0.9 | 1.9 | 3.8 | 7.2 | 8.6 | 10.4 | 11.6 | 13.7 |  |
| 12 | 1.2 | 2.6 | 5.1 | 9.5 | 11.3 | 13.6 | 15.1 | 17.8 |  |
| 15 | 1.5 | 3.2 | 6.3 | 11.8 | 13.9 | 16.7 | 18.5 | 21.7 |  |
| 18 | 1.8 | 3.8 | 7.5 | 13.9 | 16.5 | 19.7 | 21.8 | 25.5 |  |
| 21 | 2.1 | 4.4 | 8.7 | 16.1 | 18.9 | 22.5 | **24.9** | **29.0** |  |
| 24 | 2.4 | 5.1 | 9.9 | 18.1 | **21.3** | **25.3** | 28.0 | 32.4 |  |
| 27 | 2.7 | 5.7 | 11.0 | 20.1 | 23.7 | 28.0 | 30.9 | 35.7 |  |
| 30 | 3.0 | 6.3 | 12.2 | **22.1** | 25.9 | 30.6 | 33.6 | 38.7 |  |
| 33 | 3.2 | 6.9 | 13.3 | 24.0 | 28.1 | 33.1 | 36.3 | 41.7 |  |
| 36 | 3.5 | 7.5 | **14.4** | 25.9 | 30.2 | 35.5 | 38.9 | 44.5 |  |
| 39 | 3.8 | 8.1 | 15.5 | 27.7 | 32.3 | 37.8 | 41.3 | 47.1 |  |
| 42 | 4.1 | 8.7 | 16.6 | 29.5 | 34.3 | 40.0 | 43.7 | 49.6 |  |
| 45 | 4.4 | 9.3 | 17.7 | 31.3 | 36.2 | 42.2 | 45.9 | 52.0 |  |
| 48 | 4.7 | 9.9 | 18.8 | 33.0 | 38.1 | 44.2 | 48.1 | 54.3 |  |
| 51 | 5.0 | 10.5 | 19.8 | 34.6 | 40.0 | 46.2 | 50.2 | 56.5 |  |
| 54 | 5.3 | **11.0** | 20.9 | 36.2 | 41.7 | 48.2 | 52.2 | 58.6 |  |
| 57 | 5.5 | 11.6 | 21.9 | 37.8 | 43.4 | 50.0 | 54.1 | 60.6 |  |
| 60 | 5.8 | 12.2 | 22.9 | 39.3 | 45.1 | 51.8 | 56.0 | 62.5 |  |
| 63 | 6.1 | 12.8 | 23.9 | 40.8 | 46.7 | 53.5 | 57.7 | 64.3 |  |
| 66 | 6.4 | 13.3 | 24.9 | 42.3 | 48.3 | 55.2 | 59.4 | 66.0 |  |
| 69 | 6.7 | 13.9 | 25.8 | 43.7 | 49.8 | 56.8 | 61.1 | 67.6 |  |
| 72 | 6.9 | 14.4 | 26.8 | 45.1 | 51.3 | 58.4 | 62.6 | 69.1 |  |
| 75 | **7.2** | 15.0 | 27.7 | 46.5 | 52.8 | 59.8 | 64.1 | 70.6 |  |
| 78 | 7.5 | 15.5 | 28.7 | 47.8 | 54.2 | 61.3 | 65.6 | 72.0 |  |
| 81 | 7.8 | 16.1 | 29.6 | 49.1 | 55.5 | 62.7 | 66.9 | 73.4 |  |
| ^a^ Calculation of decrease of infected cells in the chemostat was performed for each dilution rate using the equation 4. The initial number of infected cell (I_0_) was set to be 3∙10^3^ PFU mL^-1^ in all cases. Numbers in bold represent approximate decrease in infected cells in our experiments for each dilution rate. | | | | | | | | | |

**Bacteriophage population growth rate as a function of dilution rate**

| Table S3. Bacteriophage population growth rate determined for different dilution rates^a^ | |
| --- | --- |
| Dilution rate (h^-1^) | Bacteriophage population growth rate (h^-1^) |
| 0.06 | 2.86 |
| 0.13 | 3.79 |
| 0.26 | 4.91 |
| 0.5 | 5.73 |
| 0.6 | 6.64 |
| 0.73 | 6.80 |
| 0.82 | 7.68 |
| 0.98 | 8.06 |
| ^a^ Bacteriophage population growth rate was calculated for each dilution rate by inserting experimentally determined burst size, latent period and adsorption constant in equation 2. | |

**RNA/protein ratio determination**

**
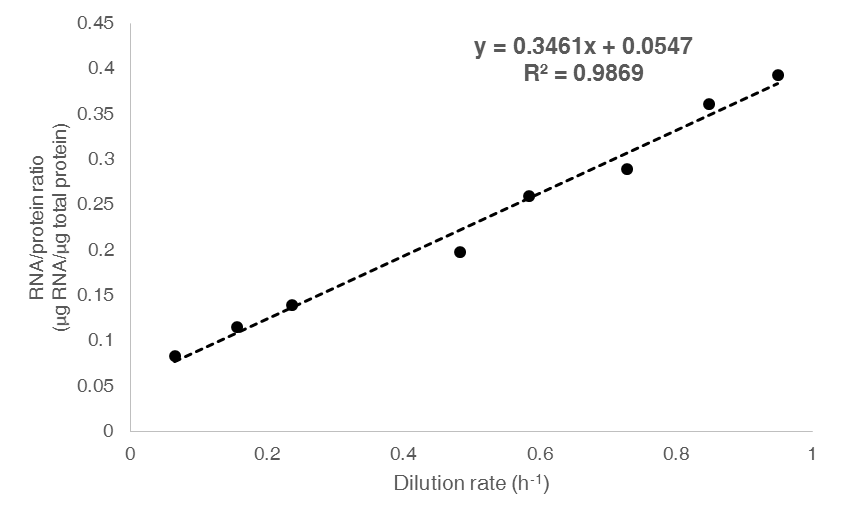
**

**Figure S2.** RNA/protein ratio as a function of dilution rate. Black dots represent obtained results, whereas dotted line represents linear correlation of data (R^2^= 0.9831).
